# Exposure to violence in public places among youths with a refugee background and their Swedish-born peers

**DOI:** 10.1038/s41598-026-56584-5

**Published:** 2026-06-05

**Authors:** Carolina Bråhn, Natalie Söderlind, Hongru Zhai, Isabel Lindbom, Johan Andersson, Laura Korhonen

**Affiliations:** 1 Barnafrid and Department of Biomedical and Clinical Sciences, Linköping University, Linköping, Sweden; 2 Centre for Social and Affective Neuroscience and Department of Biomedical and Clinical Sciences, Linköping University, Linköping, Sweden; 3 Clinical Department of Child and Adolescent Psychiatry in Linköping, 581 85 Linköping, Region Östergötland Sweden

**Keywords:** Violence in public places, Refugee background, Youth, Child participatory approach, Health care, Risk factors

## Abstract

Youths constitute a large proportion of forcibly displaced individuals worldwide. Although exposure to violence in countries of origin and during migration is well documented, less is known about its persistence in resettlement contexts, particularly regarding violence in public places (ViPP). This study aimed to examine youths’ perceived societal safety, exposure to ViPP in Sweden, and differences between participants with and without a refugee background. The study included 79 youths with a refugee background (M = 21.4 years, SD = 2.3, 54.4% male) and 105 Swedish-born peers (M = 21.5 years, SD = 2.4, 50.9% male). Data were collected between 2019 and 2022 using a questionnaire developed through a participatory approach. Fisher’s exact test was used for quantitative analyses, and content analysis was applied to qualitative data. A total of 33.3% reported experiences of ViPP in Sweden. Despite descriptive differences between groups, no statistically significant differences were observed in direct exposure to ViPP between participants with and without a refugee background. However, participants with a refugee background reported significantly higher levels of perceived unsafety and greater restrictions in their daily lives. Qualitative responses indicated that increased education, surveillance, and adult presence could improve public safety. These findings highlight the need to ensure the safety of youths in public places, particularly by addressing perceived unsafety among young people with a refugee background.

**Supplementary Information:**

The online version contains supplementary material available at 10.1038/s41598-026-56584-5.

## Introduction

Globally, it is estimated that one billion children and youths have been exposed to violence in the past year^1^, and research has shown that males are generally more likely to experience violence than females^2,3^. However, females tend to face greater exposure to domestic and sexual violence than males^4^. In addition, studies indicate that exposure to multiple forms of violence is common^4–6^, and childhood victimisation often predicts exposure to violence in adulthood^7^.

Violence has well-known negative effects on health and is considered a serious public health issue^8^. It can be lethal and is associated with various emotional and behavioural problems, including depressive symptoms, substance abuse and delinquency^7,9^. Furthermore, frequent exposure to violence over time can lead to emotional numbness towards it^10,11^. Violence also imposes high costs on society^8^.

One specific type of violence is community violence, which refers to “*incidents involving harm and threats of harm to individuals or property in neighbourhood*”^10,12^. The concepts of community violence and violence in public places overlap. Violence in public places (ViPP) extends beyond the neighbourhood, encompassing a broader range of exposures^12^. These can take direct (personal victimisation) or indirect (witnessing or hearing of violence) forms, with the latter being more common^3,9^. In 2024, 5.1% of respondents in a Swedish public health survey reported having experienced violence or threats of violence^13^. Moreover, 24% reported feeling unsafe and, consequently, refrained from going out after dark^14^.

Although we know that exposure to violence is particularly prevalent among individuals with a refugee background^15^, only a few studies have explicitly examined exposure to ViPP within this group^16,17^. Research focusing specifically on youths aged 18–25 years with a refugee background is limited and often merged with the general adult population, despite this developmental stage being a crucial transition from adolescence to adulthood, marked by changing rights^18^, variations in service provision^19^, and societal support^20^. This period also coincides with a well-documented peak in the incidence of various mental health issues^21,22^, contributing to increased vulnerability.

Previous studies have indicated that exposure to community violence among youths with a refugee background increases internalising symptoms (e.g., anxiety and depression) and externalising symptoms (e.g., delinquency and substance abuse)^16,23^. Additionally, research involving general populations with experiences of ViPP has demonstrated that males tend to report higher levels of exposure than females^3,24^, older children are generally more exposed than younger children^6,10^, and having a disability or living in poverty increases the risk of exposure^25^.

Until recently, Sweden maintained an inclusive migration policy, receiving 780,215 asylum claims from 2000 to 2021, peaking in 2015. Since then, the numbers have decreased, with 12,644 new asylum claims received in 2023 and 9,645 in 2024, respectively^26^. Between 2000 and 2021, a significant portion (45%) of all asylum seekers in Sweden, including both children and adults, originated from Syria, Afghanistan, Iraq, and Somalia. These countries remain the primary countries from which people flee^27^ .

Previous Swedish studies on youths with a refugee background have examined somatic health^28^, healthcare usage^19^, and mental health issues^29–32^, which have also been addressed in the wider Nordic literature^33,34^. However, to date, very few studies have been identified examining youth’s exposure to ViPP including those among participants with a refugee background in the country of resettlement.

To address this knowledge gap, this study aimed to examine youths’ perceived societal safety and exposure to ViPP, as well as its impact on their daily lives, and differences between participants with and without a refugee background. It also explored their suggested solutions for improving safety in public places.

## Method

### Study overview and data management

The present study is part of the ongoing research project “The Long Journey to Shelter,” which investigates mental health among adolescents and youths with a refugee background living in Sweden^35^. It employed a mixed-method design, i.e., both quantitative and qualitative data were obtained from a structured questionnaire described below, and analysed within the same study. The data were analysed using R Statistical Software (version 4.3.3)^36^.

### Ethics

The study is approved by the Swedish National Ethics Authority (2018/292-31; amendments 2018/504-32; 2020-00949; 2022-05591) and conducted in accordance with the World Medical Association Declaration of Helsinki—Ethical Principles for Medical Research Involving Human Subjects. Written informed consent was obtained from the participants (all over 18) involved in the study. Before giving consent, participants received oral (with interpreter assistance if needed) and written information about the study through a leaflet available in multiple languages. If they agreed to participate, written informed consent was obtained. Also, the adolescents involved in the cocreation workshop to develop the survey measuring violence in public places provided written informed consent after receiving oral and written information. For minors under 15 who participated in the cocreation workshop, written informed consent was also obtained from a parent or legal guardian in accordance with Swedish legislation and ethical approval.

### Participants

The inclusion criteria for participants were as follows: (a) individuals with a refugee background, defined as current or former asylum-seekers aged 18 to 25 years residing in Sweden, with no restrictions on duration of residence^35^; and (b) Swedish-born individuals aged 18 to 25 years included as a comparison group. Participants with a refugee background (n = 79) were recruited nationwide through healthcare services, social services, schools, social media, and non-governmental organisations between 2019 and 2022^30,32^. All participants were recruited by convenience sampling due to a missing sampling frame for asylum seekers and non-registered residents. Participants without a refugee background (n = 105) were then matched to the already recruited participants with a refugee background.

### Procedure

Participants interested in the study received more detailed information, both verbally and in writing, translated into their native language. Written consent was obtained from all participants. The interviews were conducted with a professional interpreter. However, most Arabic-speaking participants were interviewed by an Arabic-speaking research assistant employed within the research group. Data on exposure to ViPP was collected as part of the larger interview protocol^35^. Each interview took approximately 1.5 h to complete.

### Questionnaire on violence in public places

The questionnaire was developed using the Child Participatory Approach model^37^. Participants (n = 36) aged 13–18 years were recruited through schools, refugee centres, and Save the Children Sweden specifically for instrument development. A series of structured workshops was conducted to explore participants’ perceptions and experiences of violence in public places, as well as related needs and prevention strategies. The workshop material was analysed using qualitative content analysis, which informed the development of questionnaire items capturing key domains of interest, including direct exposure, indirect exposure (e.g., witnessing violence), perceived safety, and responses to violence. The development was primarily grounded in participants’ lived experiences; however, the identified domains are consistent with established conceptualisations of community violence exposure in previous research. The resulting questionnaire comprised a set of items reflecting these domains rather than a single underlying construct. The preliminary version was sent for review by participating youths. Although the questionnaire was developed with adolescents aged 13–18 years, it was applied to young adults aged 18–25 in the present study. While these groups share overlapping exposure contexts, developmental differences may influence how items are interpreted.

### Quantitative analysis

To improve interpretability, ordinal response categories (e.g., “very often”, “quite often”, “seldom”) were collapsed into binary variables (“yes” vs. “no”), with “never” coded as “no”. This approach was adopted to address low cell counts and enable robust statistical testing.

The questionnaire was not treated as a unified scale. Instead, individual items were analysed as separate indicators representing different dimensions of violence in public places, including personal exposure, witnessing violence, perceived safety, and behavioural adaptations.

Given the categorical nature of the data and small subgroup sizes, Fisher’s exact test was used to assess group differences, and odds ratios with 95% confidence intervals were calculated. For detailed information on the converted data, please see the Supplementary Table S1.

### Qualitative analysis

Each question on the questionnaire allowed for free-text response. The responses to the questions, “*If you are comfortable doing so, please tell us about the worst experience of violence you have had in a public place in Sweden”, “What do you think needs to be done to make your life less affected?”*, and *“Would you like to tell us more about what should be done to protect children and young people from being exposed to violence in public places?”*, were analysed using content analysis^38^ and abductive coding based on the survey framework. The respondents’ answers were not separated based on a refugee background. This was due to confidentiality issues, since very few participants in the refugee background group provided answers to the open-ended questions. Initially, CB and IL collected all meaning units, ensuring their relevance to the specific questions, and jointly coded portions of the material to maintain consistency. Subsequently, CB and IL completed the remaining coding in parallel, followed by subcategorising and categorising the codes for each question. LK reviewed the categorised data before the categories were finalised and then divided into themes by CB, which enabled a deeper interpretation of the data. These were also reviewed by LK before being finalised.

## Results

### Sample characteristics

Table 1 presents the demographic data of the sample. The study included 79 youths with a refugee background (M = 21.4 years old, SD = 2.3) and 105 Swedish-born youths (M = 21.5 years old, SD = 2.4). Gender distribution was balanced across groups. The majority of participants resided in urban municipalities, particularly among Swedish-born youths. Municipality of residence was classified using the Swedish Association of Local Authorities and Regions^39^ municipality grouping system and further categorised as urban, semi-urban, or rural. Minority group affiliation was similar in both groups but differed in nature; among youths with a refugee background, minority status was primarily related to religious beliefs, whereas among Swedish-born youths it was mainly related to sexual orientation and gender identity. Most participants were studying at the time of the interview, and mainly reported “other”, which largely reflected university level. Approximately half of the participants with a refugee background and around two-fifths of Swedish-born participants lived with their family. Parental education was categorised as low, medium, or high according to the Swedish standard education classification^40^, and education level of the mother and father was combined into a single variable based on the highest education level of either parent.

**Table 1:** Demographics of participants.

Demographics	Refugee background (*n* = *79*)	Swedish-born (*n* = *105*)
Age, M (SD)	21.4 (*2.30*)	21.5 (*2.43*)
Gender, n (%)		
Male	43 (54.4)	54 (50.9)
Female	36 (45.6)	48 (45.3)
Other	0 (0)	4 (3.8)^a^
*Missing*	*0*	*0*
Municipality of residence, n (%)		
Urban	55 (69.6)	89 (84.8)
Semi-urban	16 (20.3)	14 (13.3)
Rural	11 (13.9)	7 (6.7)
*Missing*	*0*	*0*
Minority group affiliation, yes, n (%)	24 (30.4)	36 (34.0)
*Missing*	*3*	*0*
Current education level, n (%)		
Junior High School (Year 9)	1 (1.3)	0 (0)
High School	21 (26.6)	19 (17.8)
Other^b^	41 (51.9)	38 (35.8)
*Missing*	*16*	*49*
Living with family, yes, n (%)	44 (55.7)	43 (40.6)
*Missing*	*0*	*1*
Parent status, n (%)		
Both alive	62 (78.5)	103 (98.1)
One alive	13 (16.5)	1 (0.9)
Deceased	4 (5.1)	1 (0.9)
*Missing*	*0*	*0*
Parents´ country of birth, n (%)		
Sweden	0 (0)	95 (90.5)
Syria	64 (81.0)	1 (1.0)
Other Middle Eastern countries	8 (10.1)	2 (1.9)
Other European countries	0 (0)	17 (16.2)
Asian countries	12 (15.2)	7 (6.7)
South American countries	0 (0)	3 (2.9)
African countries	0 (0)	1 (1.0)
*Missing*	*0*	*0*
Parental education level, n (%)		
High	46 (58.2)	85 (81.0)
Medium	14 (17.7)	16 (15.2)
Low	19 (24.1)	4 (3.8)
*Missing*	*0*	*0*

^a^Non-binary or gender-fluid. ^b^University, Community College, Adult High School, or Swedish for Immigrants (SFI). Parents´ level of education is based on the highest level of education between parents.

### Exposures to violence in public places

Table 2 presents frequencies on perceived societal safety, experiences and consequences of ViPP in Sweden for both study groups, while Tables 3 and 4 present the differences between the groups and between genders.

**Table 2:** Response frequencies on perceived societal safety, exposure to ViPP and adaptive behaviours.

Dependent variable	Overall (n = 184)	Refugee background (n = 79)	Swedish-born (n = 105)
Safe neighbourhood, n (%)			
Always	70 (38.0)	18 (22.8)	52 (48.6)
Most of the time	65 (35.3)	15 (19.0)	50 (48.6)
Seldom	31 (16.8)	29 (36.7)	2 (1.9)
Never	15 (8.2)	15 (19.0)	0 (0)
*Missing*	*3*	*2*	*1*
Spending time in unsafe areas, n (%)			
Never	61 (33.2)	22 (27.8)	39 (37.1)
Seldom	80 (43.5)	25 (31.6)	55 (52.4)
Quite often	22 (12.0)	12 (15.2)	10 (9.5)
Very often	5 (2.7)	4 (5.1)	1 (1.0)
*Missing*	*6*	*6*	*0*
Friend/Acquaintance exposed to violence, n (%)			
Never	65 (35.5)	35 (44.9)	30 (29.0)
Seldom	74 (40.4)	17 (21.8)	57 (54.3)
Quite often	20 (10.9)	4 (5.1)	16 (15.2)
Very often	11 (6.0)	11 (14.1)	0 (0)
*Missing*	*14*	*12*	*2*
Personally exposed to violence, n (%)			
Never	117 (63.6)	53 (67.1)	64 (60.7)
Seldom	53 (28.8)	18 (22.8)	35 (33.3)
Quite often	5 (2.7)	1 (1.3)	4 (3.8)
Very often	4 (2.2)	2 (2.5)	2 (1.9)
*Missing*	*5*	*5*	*0*
Avoiding going outside certain hours, n (%)			
No	105 (57.4)	29 (37.2)	76 (72.4)
Yes	75 (41.0)	46 (59.0)	29 (27.6)
*Missing*	*2*	*1*	*1*
Affected/restricted everyday life, n (%)			
Not at all	141 (78.8)	53 (69.7)	88 (85.7)
Sometimes	27 (15.1)	16 (21.1)	11 (10.5)
Often	7 (3.9)	5 (6.6)	2 (1.9)
Almost always	2 (1.1)	0 (0)	2 (1.9)
*Missing*	*6*	*3*	*3*
Worried family, n (%)			
Not at all	70 (38.5)	29 (38.7)	41 (39.3)
Seldom	29 (15.9)	10 (13.3)	19 (18.1)
Sometimes	35 (19.4)	7 (9.3)	28 (26.7)
Almost always	19 (10.6)	4 (5.3)	15 (14.3)
*Missing*	*5*	*4*	*1*

Data for answers “don´t know”, or “don’t want to answer” in each question are not presented.

**Table 3:** Differences between the study groups in perceived societal safety, exposure to ViPP and adaptive behaviours.

Dependent variable	Refugee Yes (%)	Swedish-born Yes (%)	Odds Ratio (*95% CI*)	*p*-value
Safe neighbourhood				
All	78.5%	99.1%	0.0 (*0.00–0.18*)	< .001
Female	66.7%	97.8%	0.0 (*0.00–0.24*)	< .001
Male	88.4%	100%	0.0 (*0.00–1.14*)	0.034
Spending time in unsafe areas				
All	51.9%	62.9%	1.37 (*0.69–2.74*)	0.422
Female	55.6%	55.4%	1.34 (*0.49–3.76*)	0.643
Male	72.1%	66.7%	1.54 (*0.57–4.34*)	0.373
Friend/Acquaintance exposed to violence				
All	41.0%	69.5%	0.38 (*0.19–0.75*)	0.004
Female	41.7%	63.8%	0.54 (*0.19–1.51*)	0.235
Male	40.5%	74.1%	0.28 (*0.10–0.74*)	0.007
Personally exposed to violence				
All	26.6%	39.0%	0.62 (*0.31–1.23*)	0.154
Female	27.8%	44.7%	0.50 (*0.17–1.37*)	0.170
Male	27.9%	33.4%	0.79 (*0.29–2.10*)	0.655
Avoiding going outside certain hours				
All	59.0%	27.6%	4.12 (*2.11–8.22*)	< .001
Female	77.8%	48.9%	3.50 (*1.27–11.11*)	0.012
Male	42.9%	9.3%	8.19 (*2.51–31.06*)	< .001
Affected/restricted everyday life				
All	27.7%	14.3%	2.31 (*1.04–5.28*)	0.036
Female	28.6%	23.4%	1.30 (*0.43–3.98*)	0.618
Male	26.8%	5.7%	6.28 (*1.49–38.02*)	0.006
Worried family				
All	54.4%	59.1%	0.98 (*0.51–1.90*)	1.000
Female	70.6%	63.8%	1.42 (*0.49–4.32*)	0.625
Male	46.3%	57.4%	0.68 (*0.27–1.68*)	0.401

Yes = response options ‘seldom’, ‘quite often’ and ‘very often’ collapsed.

**Table 4:** Differences between genders in perceived societal safety, exposure to ViPP and adaptive behaviours.

Dependent variable	Female Yes (%)	Male Yes (%)	Odds Ratio (*95% CI*)	p-value
Safe neighbourhood				
All	84.4%	94.8%	3.59 (*1.01–16.11*)	0.031
Refugee	66.7%	88.4%	4.27 (*1.1–20.53*)	0.021
Swedish-born	97.8%	100%	na	-
Spending time in unsafe areas				
All	47.0%	66.0%	1.71 (*0.87–3.38*)	0.111
Refugee	36.1%	65.1%	1.84 (*0.60–5.79*)	0.305
Swedish-born	55.4%	66.7%	1.61 (*0.67–3.92*)	0.307
Friend/Acquaintance exposed to violence				
All	54.2%	59.5%	1.19 (*0.61–2.33*)	0.633
Refugee	41.7%	40.5%	0.85 (*0.29–2.48*)	0.808
Swedish-born	63.8%	74.1%	1.63 (*0.63–4.32*)	0.278
Personally exposed to violence				
All	37.3%	29.9%	0.75 (*0.38–1.46*)	0.425
Refugee	27.8%	27.9%	0.98 (*0.32–3.07*)	1.000
Swedish-born	27.9%	33.4%	0.62 (*0.26–1.50*)	0.307
Avoiding going outside certain hours				
All	61.4%	24.0%	0.21 (*0.10–0.41*)	< .001
Refugee	77.8%	42.9%	0.25 (*0.08–0.74*)	0.009
Swedish-born	48.9%	9.3%	0.11 (*0.03–0.34*)	< .001
Affected/restricted everyday life				
All	27.7%	14.3%	2.31 (*1.04–5.28*)	0.036
Refugee	28.6%	26.8%	0.98 (*0.32–3.07*)	1.000
Swedish-born	23.4%	5.7%	0.20 (*0.03–0.85*)	0.018
Worried family				
All	49.4%	44.2%	0.55 (*0.28–1.08*)	0.083
Refugee	29.4%	26.8%	0.36 (*0.12–1.06*)	0.054
Swedish-born	63.8%	57.4%	0.75 (*0.31–1.83*)	0.539

na = not applicable. Yes = response options ‘seldom’, ‘quite often’ and ‘very often’ collapsed.

Personal experiences of physical, psychological, or sexual violence in a public place in Sweden were reported by 33.3% of the participants. No statistically significant differences were observed between the study groups (OR = 0.62, 95% CI [0.31–1.23], *p* = 0.154). More than half of the participants reported that a friend or acquaintance had experienced such violence in Sweden, and this was significantly more commonly reported among Swedish-born participants (OR = 0.38, 95% CI [0.19–0.75], *p* = 0.004). Among those exposed to physical, psychological or sexual ViPP, experiences of public humiliation, witnessing someone unknown be subjected to violence in a public place, and verbal violence were the most frequently reported types of violence, where public humiliation was mainly related to either ethnicity and/or clothing, or their sex and/or appearance. There was no significant difference between the study groups in any type of violence experienced. However, significantly more women than men reported experiences of verbal (OR = 0.20, 95% CI [0.03–0.98], *p* = 0.044) and sexual violence (OR = 0.19, 95% CI [0.04–0.79], *p* = 0.018) across both study groups. For more detailed descriptions of types of violence, see the Supplementary Table S2 to S5.

The most common places to be exposed to violence were in park-like areas or on school grounds, whereas residential areas, shopping centres, and restaurants were the least common. Perpetrators were usually unknown individuals, whereas family members and current or former partners were the least common.

The content analysis of respondents’ worst experiences of ViPP resulted in three themes, all of which are reflected in the findings from the quantitative analyses of experiences of ViPP, presented above. For details on the categories and themes from analysing participants’ worst experiences, see the Supplementary Fig. S1.

### Feeling unsafe and adapted behaviours.

Significantly more participants with a refugee background, than Swedish-born participants, reported that it is not safe for children and young people to move around in their residential area (OR = 0.0, 95% CI [0.00–0.18], *p* < 0.001), and indicated that they avoided going outside during certain hours of the day (OR = 4.12, 95% CI [2.11–8.22], *p* < 0.001), particularly after dark or during specific times such as weekends or school hours. Likewise, it was significantly more common among women than men to perceive that their residential area was not safe (OR = 3.59, 95% CI [1.01–16.11], *p* = 0.031), and to avoid going outside certain hours during the day (OR = 0.21, 95% CI [0.10–0.41], *p* < 0.001). This was also significantly different within and between the study groups by gender (see Tables 3–4 and for a more detailed description of differences in response frequencies between genders, please see the Supplementary Table S6). Conversely, Swedish-born participants reported spending more time in unsafe areas compared to their peers with a refugee background, though with no significant differences (OR = 1.37, 95% CI [0.69–2.74], *p* = 0.422).

A quarter of all participants reported that their daily lives were affected by violence or threats of ViPP, more so among participants with a refugee background compared to Swedish-born individuals (OR = 2.31, 95% CI [1.04–5.28], *p* = 0.036), and more among females than males (OR = 2.31, 95% CI [1.04–5.28], *p* = 0.036). Both study groups reported restrictions in the places they chose to visit, and participants with a refugee background also indicated that certain activities were restricted by the time of day. In contrast, Swedish-born participants reported restrictions on whether they engaged in activities alone or with others.

Approximately half of the participants reported that their families were concerned about the risk of exposure to violence outside their homes, with no significant differences between the study groups (OR = 0.98, 95% CI [0.51–1.90], *p* = 1.000).

### Areas of improvement for safe public places

Table 5 shows the results of the content analysis of participants’ suggestions on areas of improvement. Five themes were identified:

**Table 5:** Descriptions of categories and themes from participants improvement suggestions.

Themes	Categories	Citations
Proper education on violence and support	We need to understand each other´s perspectives	“Force people who use violence against others to get to know other people [victims] who are not like them … so that people [offenders] can understand the difficulties they [victims] have been through.”
	Information and education	“Better education about consent, a societal change.”
	Give more education about violence and the society	“Do activities for children and teach them about the dangers of violence … It is also important to teach children now that violence is dangerous in order to prevent them from being violent in the future…”
	Knowledge and education	“Talk more about violence in school. There is a lot of violence on social media – good cooperation between schools and parents is needed … Start talking early on about why it is not okay.”
	Increased inclusion	“Reduce exclusion, reduce socio-economic differences.”
Stricter sentences and a greater police presence	Change in law and order	“At present, the penalties for serious crimes such as rape are very mild, so it is too easy to get away with them … Certain crimes should carry harsher penalties and be easier to convict for.”
	Improve community support and safety	“… presence of police patrols in the city and in public places is extremely important and gives both young people and adults a sense of security.”
	Change in laws and regulations	“… it’s important that there are consequences for those who bully or use violence [in school] … Now, kids are learning that violence often has no consequences.”
Thoughtful and security-oriented urban planning	Change in social structures	“Work preventively so that people don’t end up in trouble, because delinquency is linked to that kind of thing…”
	Create a safer community	“More surveillance seems like a good solution.”
	Thoughtful construction/infrastructure	“… the places we are in are designed in a way that is conducive to violence – [lack of] lights, secluded places. It is possible to build places that do not encourage violence.”
The government’s responsibility for social safety	The school takes responsibility	“The school needs to create a safe environment…”
	Preventively work	“Preventive work, rather than subsequent interventions that restricts the freedom of children and young people.”
	Resources for order and security	“Some more surveillance … cameras are now going to be installed there, which I think is a good thing when it’s not possible to have police on site all the time …”
	Functioning help- and support system	“One should make sure that children know where to turn if something happens, and try to counteract stigma.”
Adults’ responsibility for a safe childhood	Respect children´s rights	“… children need to have a private life [for parents]”
	Adults must take responsibility and instil safety	“Parents [or] families must teach their children how to behave outside the home … it is very important that children behave themselves.”
	Safe adults outside	“Having more adult presence. The outreach team. Safety patrols, more sober adults out and about.”
	Create safe places and meaningful activities for children and young people	“The most important preventive measure is to provide meaningful leisure activities and support to those who victimise others… “
	People must dare to act	“More communication, take more action, noticed that adults tend to ignore things, e.g. as soon as it is outside school.”
	That adults get involved with children and young people	“Teachers and other adults should be more present and take situations seriously. Adults must dare to speak up and be attentive and also discuss at school what is acceptable behaviour and what is not, e.g. consent for sexual acts.”

Two questions are aggregated in this table: (1) What do you think needs to be done to make your life less affected? (2) Would you like to tell us more about what should be done to protect children and young people from being exposed to violence in public places?

*Proper education on violence and support*. Respondents emphasised the importance of education regarding consent, societal norms, prejudices, respectful behaviours, different forms of violence, and the dangers related to violent behaviours. They suggested that discussions about violence could raise awareness and improve information provided about child protective services, which was also noted regarding the police. Many respondents described how misinformation spreads easily, potentially causing distrust within the community. Additionally, there was a call for more accessible information on how to seek support.

*Stricter sentences and increased police presence.* Respondents suggested enforcing harsher penalties and stricter laws to protect children and young people, highlighting the need to address the inadequate responses to violent behaviours in schools. They expressed distrust in the police’s commitment to addressing reported violence but believed that a greater police presence could improve community safety and, consequently, foster trust in law enforcement. Additionally, respondents recommended stricter regulations on drug and alcohol use, as well as children’s use of social media.

*Thoughtful and security-oriented urban planning*. Respondents argued that improved infrastructure, more streetlights, and enhanced adult supervision could help prevent violence by increasing visibility. The idea of camera surveillance was proposed, but opinions varied. While some considered it a reasonable solution, others saw it as a violation of privacy, although they recognised that it might still be necessary. Additional suggestions included better public transport.

*The government’s responsibility for social safety.* Respondents highlighted that political decisions, methods, approaches, and routines within schools, infrastructure, and healthcare can influence preventative efforts to reduce exposure to violence. They suggested increased surveillance by responsible adults, such as security personnel, social workers, teachers, and healthcare professionals. Additionally, respondents recommended making help and support more accessible for both victims and perpetrators. They noted that the involvement of engaged adults may lead to a better understanding of the support needs of children and young people, thereby enabling them to receive appropriate assistance.

*Adults’ responsibility for a safe childhood*. Respondents emphasised the role and responsibility of parents in their children’s education regarding what constitutes good behaviour and in creating safe environments. They highlighted the perceived lack of moral courage among adults and the need for their action. Another important protective factor mentioned was extracurricular activities, which may provide safe places for children and young people, helping keep them away from negative influences.

## Discussion

The aim of this study was to examine youths’ perceived societal safety, exposure to ViPP in Sweden, its impact on their daily lives, and differences by refugee background. Similar to previous studies^2,23,41^, 33.3% of all participants had personally experienced ViPP in Sweden. The Swedish-born youths more frequently reported having a friend/acquaintance exposed to violence compared to youths with a refugee background. A plausible explanation is that perceptions of violence may differ between the groups or reflect the responders and their friends´ and acquaintances´ minority identities. These differences may also entail distinct forms of stigma and vulnerability in public places, which could influence both exposure to violence and perception of safety. Another explanation may relate to the sample characteristics and methodology, which will be addressed in the limitations section. The findings also suggest that, in contrast with previous research^2,3^, a higher proportion of females than males have been exposed to violence. However, in line with existing evidence^4^, the finding showed that women have been exposed to sexual violence to a greater extent than men.

Data were partially collected during the COVID-19 pandemic, which may have influenced reporting as the number of crimes and concerns about being exposed to crime decreased slightly from 2020 onwards^14,42^, even though lifetime exposures in Sweden were investigated. However, in recent years, Swedish society has experienced an unprecedented increase in shootings and explosions in public places^43^. Moreover, concerns about crime in society have increased from 43% of the Swedish population in 2019 to 50% today^14^. This warrants longitudinal follow-up of experiences of ViPP.

Many participants, especially youths with a refugee background, reported high levels of perceived unsafety. Youths with a refugee background viewed their neighbourhoods as less safe, avoided going out more, and reported greater restrictions in their everyday lives due to ViPP than Swedish-born peers. Feelings of unsafety and heightened awareness of potential threats may reflect mental and emotional challenges, including trauma-related symptoms, which are common among young people with a refugee background^24,30^. These differences should not be interpreted as reflecting inherent group characteristics, but rather as shaped by contextual, social, and experiential factors influencing how safety is perceived and navigated in everyday life.

The relationship between neighbourhood context and perceived safety may also be influenced by these broader contextual and individual factors. In the present sample, youths with a refugee background were more likely to reside in semi-urban and rural areas compared to Swedish-born peers, suggesting that perceived unsafety reflects broader social and experiential influences rather than an urban neighbourhood context alone. Such perceptions may lead youths to avoid public places, potentially increasing the risk of social isolation, which can complicate integration for newly arrived individuals with a refugee background.

Although detailed data on socioeconomic status were not collected, several indicators were included. Parental education, living arrangements, and municipality type provide partial insight into participants socioeconomic and structural context. These factors may be particularly relevant when interpreting perceived safety and daily life restrictions in public places. However, more specific residential-level data are important for future research, as the prevalence of ViPP may differ between residential areas within different municipalities, potentially influencing both exposure to ViPP and perception of societal safety.

This study also aimed to investigate the participants’ views on necessary measures to mitigate exposure to ViPP in Sweden. Participants’ suggestions for improvement aligned with previous research. As noted by participants, extracurricular activities can strengthen peer bonds and provide meaningful engagement, thereby reducing externalising problems^44^, particularly for youth with a refugee background who often face an increased risk of separation from friends and family^18^. Participants also emphasised the role of parents in ensuring children’s safety. Prior research demonstrates that a positive parent–child relationship is associated with less exposure to violence in general, reduced aggressive behaviours^16^, and improved academic performance^24^. As indicated by participants and consistent with previous studies^44^, municipalities bear primary responsibility for community safety. This entails ensuring urban safety through adequate lighting and surveillance and involving adults, such as teachers and social workers^44^. Participants also emphasised the importance of increased police presence and clearer legal consequences for violent behaviour, which they perceived as important for improving safety in public places. Schools, for example, play a protective role against violence and can promote well-being among young people with a refugee background^18^. A notable theme among the responses was the focus on education. With comprehensive education about violence, victimisation, rights, and support services, children and youths may have a greater chance of growing up free from violence, thereby enhancing their mental and emotional well-being.

Tackling ViPP is a major concern for public health policy, as it has serious effects on physical and mental well-being^16,23^. It has also been shown to substantially impact the sense of safety in public settings and interpersonal interactions^14^. Understanding the magnitude and consequences of ViPP is important for developing public health interventions and preventive measures.

Overall, the findings underline the importance of social and structural context for youths’ perceived safety and adaptive behaviours in public places. This points to the need to distinguish between exposure to violence and its subjective and behavioural consequences. By demonstrating that exposure to violence in public places may not differ significantly between groups while perceptions and behavioural responses do, this study contributes to a more nuanced understanding of how violence is experienced by young people in resettlement contexts. These findings have important implications for prevention efforts and policy interventions, suggesting that improving perceived safety and reducing the everyday impact of violence requires not only addressing direct exposure, but also strengthening social environments, increasing adult presence, and enhancing access to information, support, and protective structures for young people.

### Strengths and limitations

This study has several strengths as well as limitations. A key strength is its focus on violence in public places among young adults, an age group that is often merged with broader age categories in previous research. Another strength is the use of a questionnaire developed through a child participatory approach, ensuring that the measures were grounded in the lived experiences of the target population.

However, several limitations should be considered when interpreting the findings. First, the questionnaire was developed with adolescents aged 13–18 years but applied to young adults aged 18–25 years, which may affect its developmental and contextual validity. Differences in life stage, autonomy, and exposure contexts may influence how questions are interpreted, and thus how well the instrument captures comparable constructs across age groups. Second, the measurement approach relied on multiple single-item indicators representing different dimensions of violence in public places rather than a validated composite scale. While this enabled a nuanced assessment of different aspects of exposure and perceived safety, it may limit comparability with other studies and reduce precision in capturing the underlying construct. Third, the relatively small sample size, particularly in subgroup analyses, may have limited statistical power. This may have reduced the ability to detect statistically significant differences between groups despite observable descriptive variation in some outcomes. Fourth, the sample was based on convenience recruitment, which limits generalisability. It is also possible that particularly vulnerable individuals were underrepresented, introducing potential bias in the findings. Finally, the data were collected between 2019 and 2022, thus limiting the findings to youths with a refugee background residing in Sweden during this time period. Collected data were also part of a longer interview protocol, including several questions about other forms of violence^35^, which may have caused fatigue, explaining missing data, especially among participants with a refugee background. These limitations should be considered when interpreting comparisons between groups.

### Future research

As one of the first studies to examine ViPP among youths in a Nordic context, this study provides contextually grounded insights through a mixed-method and participatory design. By addressing an understudied context, it contributes to an emerging field and offers a foundation for future comparative and longitudinal research. Building on these findings, future research should further investigate patterns and trends of exposure to ViPP, as well as their impact on perceived safety, cognition, and behaviour. It would also be valuable to explore how prior experiences, such as domestic violence or bullying, may influence public encounters, and how experiences of ViPP vary across neighbourhoods and urban contexts. Future research should also examine how violence in public places differs from other forms of victimisation, to determine whether it represents a distinct exposure context with unique implications for health and behaviour. Finally, a participatory approach appears particularly promising for advancing this field—not only in addressing key research questions and supporting interpretation of findings, but also in developing context-specific strategies to prevent exposure and promote societal safety among youths.

### Conclusion

This study found that one-third of the participants had experienced ViPP in Sweden, and that many, particularly those with a refugee background, felt unsafe. This may have implications for their integration into society. Participants highlighted several potential improvements, including education on violence, clearer penalties, increased police presence, surveillance, and expanded youth activities to promote safer communities.

## Supplementary Information

Below is the link to the electronic supplementary material.

Supplementary Material 1

## Data Availability

The collected dataset from the present study is not publicly available due to ethical restrictions but is available from the corresponding author on reasonable request.
