# Supplementary material for: Exposure to violence in public places among youths with a refugee background and their Swedish-born peers
Source: Sci Rep. 2026 Jun 5;16:17529. doi: 10.1038/s41598-026-56584-5 (PMC13241509; doi:10.1038/s41598-026-56584-5)
Supplement: Supplementary file 1 — Supplementary Material 1 [file 41598_2026_56584_MOESM1_ESM.docx]

**Exposure to violence in public places among youths with a refugee background and their Swedish-born peers**

**Authors:** Carolina Bråhn, Natalie Söderlind, Hongru Zhai, Isabel Lindbom, Johan Andersson and Laura Korhonen

**Supplementary materials**

| **Table S1** Description of collapsed levels for response options | |
| --- | --- |
| **Levels Before Collapsing** | **Levels After Collapsing** |
| **Is it safe for children and young people to move around in the area where you live?** | |
| Always | Yes |
| Most of the time |  |
| Seldom |  |
| Never | No |
| Don´t want to answer | (Omitted) |
| Don´t know |  |
| **Do you happen to stay in other areas where children and young people are not safe?** | |
| Never | No |
| Seldom | Yes |
| Quiet often |  |
| Very often |  |
| Don´t know | (Omitted) |
| Don´t want to answer |  |
| **Does it happen that you avoid going out because you are afraid of being assaulted, robbed or otherwise exposed?** | |
| Never | No |
| Seldom | Yes |
| Quite often |  |
| Very often |  |
| Don´t know | (Omitted) |
| Don´t want to answer |  |
| **Do you avoid going outside certain hours during the day due to fear of being exposed to violence?** | |
| No | No |
| Yes | Yes |
| Don´t know | (Omitted) |
| Don´t want to answer |  |
| **As far as you know, has a friend or acquaintance of yours ever been subjected to physical, psychological or sexual violence in a public place in Sweden?** | |
| Never | No |
| Seldom | Yes |
| Quite often |  |
| Very often |  |
| Don´t know | (Omitted) |
| Don´t want to answer |  |
| **Have you ever experienced physical, psychological or sexual violence in a public place in Sweden?** | |
| Never | No |
| Seldom | Yes |
| Quite often |  |
| Very often |  |
| Don´t know | (Omitted) |
| Don´t want to answer |  |
| **Is your everyday life affected or restricted due to violence or risk of violence?** | |
| No, not at all | No |
| Sometimes | Yes |
| Often |  |
| Almost always |  |
| Don´t know | (Omitted) |
| Don´t want to answer |  |
| **Does it happen that someone in your family is worried that you will be exposed to violence when you are outside the home?** | |
| No, not at all | No |
| Seldom | Yes |
| Sometimes |  |
| Almost always |  |
| Don´t know | (Omitted) |
| Don´t want to answer |  |
| **In a public place in Sweden, have you even experience any of the following? *** | |
| Never | No |
| Seldom | Yes |
| Quite often |  |
| Very often |  |
| Don´t know | (Omitted) |
| Don´t want to answer |  |
| Note: *The question is a multiple-choice question and refers to different types of violence; Physical violence, Sexual violence, psychological violence, verbal violence, witnessing violence against unknown person, witnessing violence against known person, other forms of violence, and publicly humiliated. | |

| **Table S2** Response frequencies of different types of violence. | | | |
| --- | --- | --- | --- |
| **Type of violence** | **Overall, n (%)** | **Refugee, n (%)** | **Swedish-born, n (%)** |
| **Physical violence** | n=55 | n=14 | n=41 |
| Never | 25 (45.4) | 5 (35.7) | 20 (48.8) |
| Seldom | 28 (50.9) | 8 (57.1) | 20 (48.8) |
| Quite often | 0 (0) | 0 (0) | 0 (0) |
| Very often | 0 (0) | 0 (0) | 0 (0) |
| *Missing* | *7* | *7* | *0* |
| **Sexual violence** | n=54 | n=13 | n=41 |
| Never | 35 (64.8) | 9 (69.2) | 26 (63.4) |
| Seldom | 16 (29.6) | 3 (23.1) | 13 (31.7) |
| Quite often | 2 (3.7) | 0 (0) | 2 (4.9) |
| Very often | 0 (0) | 0 (0) | 0 (0) |
| *Missing* | *8* | *8* | *0* |
| **Psychological violence** | n=55 | n=14 | n=41 |
| Never | 19 (34.5) | 2 (14.3) | 17 (41.5) |
| Seldom | 24 (43.6) | 7 (50.0) | 17 (41.5) |
| Quite often | 8 (14.5) | 3 (21.4) | 5 (12.2) |
| Very often | 2 (3.6) | 0 (0) | 2 (4.9) |
| *Missing* | *7* | *7* | *0* |
| **Verbal violence** | n=55 | n=14 | n=41 |
| Never | 12 (21.8) | 4 (28.6) | 8 (19.5) |
| Seldom | 25 (45.5) | 2 (14.3) | 23 (56.1) |
| Quite often | 13 (23.6) | 5 (35.7) | 8 (19.5) |
| Very often | 2 (3.6) | 0 (0) | 2 (4.9) |
| *Missing* | *7* | *7* | *0* |
| **Witnessing violence against someone close** | n=56 | n=15 | n=41 |
| Never | 27 (48.2) | 7 (46.7) | 20 (48.8) |
| Seldom | 23 (41.1) | 6 (40.0) | 17 (41.5) |
| Quite often | 4 (7.1) | 0 (0) | 4 (9.8) |
| Very often | 0 (0) | 0 (0) | 0 (0) |
| *Missing* | *6* | *6* | *0* |
| **Witnessing violence against unknown person** | n=55 | n=14 | n=41 |
| Never | 15 (27.3) | 1 (7.1) | 14 (34.1) |
| Seldom | 32 (58.2) | 9 (64.3) | 23 (56.1) |
| Quite often | 5 (9.1) | 2 (14.3) | 3 (7.3) |
| Very often | 1 (1.8) | 0 (0) | 1 (2.4) |
| *Missing* | *7* | *7* | *0* |
| **Other types of violence** | n=50 | n=14 | n=36 |
| Never | 43 (86.0) | 12 (85.7) | 31 (86.1) |
| Seldom | 2 (4.0) | 0 (0) | 2 (5.6) |
| Quite often | 1 (2.0) | 0 (0) | 1 (2.8) |
| Very often | 2 (4.0) | 2 (14.3) | 0 (0) |
| *Missing* | *12* | *7* | *5* |
| **Publicly humiliated** | n=58 | n=16 | n=42 |
| Never | 12 (20.7) | 2 (12.5) | 10 (23.8) |
| Seldom | 29 (50.0) | 8 (50.0) | 21 (50.0) |
| Quite often | 13 (22.4) | 3 (18.8) | 10 (23.8) |
| Very often | 4 (6.9) | 3 (18.8) | 1 (2.4) |
| *Missing* | *6* | *6* | *0* |
| Note: Data is calculated based on the number of participants answering “Yes” to question 7. Data for answers “don´t know”, or “don’t want to answer” in each question are not presented. | | | |

| **Table S3** Response frequencies of different types of violence stratified by gender. | | | | |
| --- | --- | --- | --- | --- |
| **Type of violence** | **Refugee background** | | **Swedish-born** | |
|  | **Female, n (%)** | **Male, n (%)** | **Female, n (%)** | **Male, n (%)** |
| **Physical violence** | n=8 | n=6 | n=21 | n=18 |
| Never | 3 (37.5) | 2 (33.3) | 12 (57.1) | 6 (33.3) |
| Seldom | 5 (62.5) | 3 (50.0) | 8 (38.1) | 12 (66.7) |
| Quite often | 0 (0) | 0 (0) | 0 (0) | 0 (0) |
| Very often | 0 (0) | 0 (0) | 0 (0) | 0 (0) |
| *Missing* | *2* | *5* | *0* | *0* |
| **Sexual violence** | n=7 | n=6 | n=21 | n=18 |
| Never | 4 (57.1) | 5 (83.3) | 9 (42.9) | 15 (83.3) |
| Seldom | 2 (28.6) | 1 (16.7) | 10 (47.6) | 3 (16.7) |
| Quite often | 0 (0) | 0 (0) | 2 (9.5) | 0 (0) |
| Very often | 0 (0) | 0 (0) | 0 (0) | 0 (0) |
| *Missing* | *3* | *5* | *0* | *0* |
| **Psychological violence** | n=8 | n=6 | n=21 | n=18 |
| Never | 1 (12.5) | 1 (16.7) | 9 (42.9) | 7 (38.9) |
| Seldom | 3 (37.5) | 4 (66.7) | 7 (33.3) | 9 (50.0) |
| Quite often | 3 (37.5) | 0 (0) | 3 (14.3) | 2 (11.1) |
| Very often | 0 (0) | 0 (0) | 2 (9.5) | 0 (0) |
| *Missing* | *2* | *5* | *0* | *0* |
| **Verbal violence** | n=8 | n=6 | n=21 | n=18 |
| Never | 1 (12.5) | 3 (50.0) | 2 (9.5) | 6 (33.3) |
| Seldom | 1 (12.5) | 1 (16.7) | 13 (61.9) | 8 (44.4) |
| Quite often | 4 (50.0) | 1 (16.7) | 5 (23.8) | 3 (16.7) |
| Very often | 0 (0) | 0 (0) | 1 (4.8) | 1 (5.6) |
| *Missing* | *2* | *5* | *0* | *0* |
| **Witnessing violence against someone close** | n=8 | n=7 | n=21 | n=18 |
| Never | 4 (50.0) | 3 (42.9) | 11 (52.4) | 8 (44.4) |
| Seldom | 3 (37.5) | 3 (42.9) | 6 (28.6) | 10 (55.6) |
| Quite often | 0 (0) | 0 (0) | 4 (19.0) | 0 (0) |
| Very often | 0 (0) | 0 (0) | 0 (0) | 0 (0) |
| *Missing* | *2* | *4* | *0* | *0* |
| **Witnessing violence against unknown person** | n=8 | n=6 | n=21 | n=18 |
| Never | 0 (0) | 1 (16.7) | 9 (42.9) | 5 (27.8) |
| Seldom | 6 (75.0) | 3 (50.0) | 8 (38.1) | 13 (72.2) |
| Quite often | 1 (12.5) | 1 (16.7) | 3 (14.3) | 0 (0) |
| Very often | 0 (0) | 0 (0) | 1 (4.8) | 0 (0) |
| *Missing* | *2* | *5* | *0* | *0* |
| **Other types of violence** | n=8 | n=6 | n=20 | n=14 |
| Never | 7 (87.5) | 5 (83.3) | 18 (90.0) | 11 (78.6) |
| Seldom | 0 (0) | 0 (0) | 0 (0) | 2 (14.3) |
| Quite often | 0 (0) | 0 (0) | 1 (5.0) | 0 (0) |
| Very often | 1 (12.5) | 1 (16.7) | 0 (0) | 0 (0) |
| *Missing* | *2* | *5* | *1* | *4* |
| **Publicly humiliated** | n=8 | n=7 | n=21 | n=18 |
| Never | 1 (12.5) | 1 (14.3) | 2 (9.5) | 8 (44.4) |
| Seldom | 4 (50.0) | 4 (57.1) | 13 (61.9) | 6 (33.3) |
| Quite often | 0 (0) | 2 (28.6) | 5 (23.8) | 4 (22.2) |
| Very often | 3 (37.5) | 0 (0) | 1 (4.8) | 0 (0) |
| *Missing* | *2* | *4* | *0* | *0* |
| Note: Data is calculated based on the number of participants answering “Yes” to question 7. Data for answers “don´t know”, or “don’t want to answer” in each question are not presented. | | | | |

| **Table S4** Differences between groups in experiences of types of violence. | | | | |
| --- | --- | --- | --- | --- |
| **Dependent variable** | **Refugees**  **Yes (%)** | **Swedish-born**  **Yes (%)** | **Odd Ratio**  **(*95% CI*)** | **p-value** |
| **Physical violence** | | | | |
| All | 27.6 % | 48.8 % | 1.59 (*0.38-7.31*) | 0.536 |
| Female | 50.0 % | 38.1 % | 2.42 (*0.35-20.22*) | 0.410 |
| Male | 25.0 % | 66.7 % | 0.76 (*0.07-11.43*) | 1.000 |
| **Sexual violence** | | | | |
| All | 10.3 % | 36.6 % | 0.58 (*0.09-2.84*) | 0.730 |
| Female | 20.0 % | 57.1 % | 0.39 (*0.03-3.43*) | 0.385 |
| Male | 8.3 % | 16.7 % | 1.00 (*0.02-16.30*) | 1.000 |
| **Psychological violence** | | | | |
| All | 34.5 % | 58.5 % | 3.47 (*0.62-36.51*) | 0.174 |
| Female | 60.0 % | 57.1 % | 4.29 (*0.40-229.27*) | 0.362 |
| Male | 33.3 % | 61.1 % | 2.45 (*0.19-142.86*) | 0.621 |
| **Verbal violence** | | | | |
| All | 24.1 % | 80.5 % | 0.43 (*0.08-2.51*) | 0.253 |
| Female | 50.0 % | 90.5 % | 0.54 (*0.02-37.20*) | 0.545 |
| Male | 16.7 % | 66.7 % | 0.35 (*0.02-3.95*) | 0.343 |
| **Witnessing violence against someone close** | | | | |
| All | 20.7 % | 51.2 % | 0.82 (*0.19-3.42)* | 1.000 |
| Female | 30.0 % | 47.6 % | 0.83 (*0.10-6.33*) | 1.000 |
| Male | 25.0 % | 55.6 % | 0.81 (*0.08-7.78*) | 1.000 |
| **Witnessing violence against unknown person** | | | | |
| All | 37.9 % | 65.9 % | 5.56 (*0.68-262.41*) | 0.144 |
| Female | 70.0 % | 57.1 % | na | - |
| Male | 33.3 % | 72.2 % | 1.51 (*0.11-90.66*) | 1.000 |
| **Other types of violence** | | | | |
| All | 6.9 % | 7.3 % | 1.70 (*0.13-16.88*) | 0.621 |
| Female | 10.0 % | 4.8 % | 2.47 (*0.03-212.43*) | 0.513 |
| Male | 8.3 % | 11.1 % | 1.09 (*0.02-26.08*) | 1.000 |
| **Publicly humiliated** | | | | |
| All | 44.8 % | 75.6 % | 2.07 (*0.36-22.05*) | 0.481 |
| Female | 70.0 % | 90.5 % | 0.75 (*0.03-49.61*) | 1.000 |
| Male | 50.0 % | 55.6 % | 4.54 (*0.41-247.51*) | 0.355 |
| Note: na = not applicable.  Yes = response options ‘seldom’, ‘quite often’ and ‘very often’ collapsed.  Data is only caluculated based on the number of participants answereing “Yes” to question 7. | | | | |

| **Table S5** Differences between gender in experiences of types of violence. | | | | |
| --- | --- | --- | --- | --- |
| **Dependent variable** | **Female**  **Yes (%)** | **Male**  **Yes (%)** | **Odd Ratio**  **(*95% CI*)** | **p-value** |
| **Physical violence** | | | | |
| All | 41.9 % | 50.0 % | 2.13 (*0.61-7.89*) | 0.259 |
| Refugee | 50.0 % | 25.0 % | 0.91 (*0.06-17.16*) | 1.000 |
| Swedish-born | 38.1 % | 66.7 % | 2.91 (*0.67-13.98*) | 0.120 |
| **Sexual violence** | | | | |
| All | 45.2 % | 13.3 % | 0.19 (*0.04-0.79*) | 0.018 |
| Refugee | 20.0 % | 8.3 % | 0.43 (*0.01-11.32*) | 1.000 |
| Swedish-born | 57.1 % | 16.7 % | 0.16 (*0.02-0.80*) | 0.020 |
| **Psychological violence** | | | | |
| All | 58.1 % | 50.0 % | 1.04 (*0.28-3.90*) | 1.000 |
| Refugee | 60.0 % | 33.3 % | 0.60 (*0.01-65.25*) | 1.000 |
| Swedish-born | 57.1 % | 61.1 % | 1.17 (*0.27-5.16*) | 1.000 |
| **Verbal violence** | | | | |
| All | 77.4 % | 46.7 % | 0.20 (*0.03-0.98*) | 0.044 |
| Refugee | 50.0 % | 16.7 % | 0.16 (*0.00-3.50*) | 0.242 |
| Swedish-born | 90.5 % | 66.7 % | 0.22 (*0.02-1.49*) | 0.112 |
| **Witnessing violence against someone close** | | | | |
| All | 41.9 % | 43.3 % | 1.36 (*0.40-4.68*) | 0.781 |
| Refugee | 30.0 % | 25.0 % | 1.30 (*0.09-19.13*) | 1.000 |
| Swedish-born | 47.6 % | 55.6 % | 1.36 (*0.33-5.85*) | 0.751 |
| **Witnessing violence against unknown person** | | | | |
| All | 61.3 % | 56.7 % | 1.33 (*0.34-5.59*) | 0.761 |
| Refugee | 70.0 % | 33.3 % | 0.00 (*0.00-27.86*) | 0.417 |
| Swedish-born | 57.1 % | 72.2 % | 1.92 (*0.43-9.55*) | 0.504 |
| **Other types of violence** | | | | |
| All | 6.5 % | 10.0 % | 2.30 (*0.24-30.39*) | 0.635 |
| Refugee | 10.0 % | 8.3 % | 1.37 (*0.01-125.24*) | 1.000 |
| Swedish-born | 4.8 % | 11.1 % | 3.15 (*0.15-203.30*) | 0.551 |
| **Publicly humiliated** | | | | |
| All | 83.9 % | 53.3 % | 0.21 (*0.03-1.01*) | 0.462 |
| Refugee | 70.0 % | 50.0 % | 0.87 (*0.01-78.32*) | 1.000 |
| Swedish-born | 90.5 % | 55.6 % | 0.14 (*0.01-0.88*) | 0.025 |
| Note: Yes = response options ‘seldom’, ‘quite often’ and ‘very often’ collapsed.  Data is only caluculated based on the number of participants answereing “Yes” to question 7. | | | | |

| **Table S6** Response frequencies on perceived societal safety, exposure to ViPP and adaptive behaviours stratified by gender. | | | | |
| --- | --- | --- | --- | --- |
| **Dependent variable** | **Refugee (n=79)** | | **Swedish-born (n=105)** | |
|  | **Female (n=36)** | **Male (n=43)** | **Female (n=47)** | **Male (n=54)** |
| **Safe neighbourhood, n (%)** | | | | |
| Always | 5 (13.9) | 13 (30.2) | 16 (34.0) | 33 (61.1) |
| Most of the time | 4 (11.1) | 11 (25.6) | 30 (63.8) | 19 (35.2) |
| Seldom | 15 (41.7) | 14 (32.6) | 0 (0) | 2 (3.7) |
| Never | 11 (30.6) | 4 (9.3) | 0 (0) | 0 (0) |
| *Missing* | *0* | *0* | *1* | *0* |
| **Spending time in unsafe areas, n (%)** | | | | |
| Never | 12 (33.3) | 10 (23.3) | 21 (44.7) | 18 (33.3) |
| Seldom | 10 (27.8) | 15 (34.9) | 24 (51.1) | 27 (50.0) |
| Quite often | 3 (8.3) | 9 (20.9) | 2 (4.3) | 8 (14.8) |
| Very often | 0 (0) | 4 (9.3) | 0 (0) | 1 (1.9) |
| *Missing* | *0* | *0* | *1* | *0* |
| **Friend/Acquaintance exposed to violence, n (%)** | | | | |
| Never | 15 (41.7) | 20 (47.6) | 16 (34.0) | 13 (24.1) |
| Seldom | 6 (16.7) | 11 (26.2) | 18 (38.3) | 36 (66.7) |
| Quite often | 2 (5.6) | 2 (4.8) | 12 (25.5) | 4 (7.4) |
| Very often | 7 (19.4) | 4 (9.5) | 0 (0) | 0 (0) |
| *Missing* | *0* | *1* | *1* | *1* |
| **Personally exposed to violence, n (%)** | | | | |
| Never | 25 (69.4) | 28 (65.1) | 26 (55.3) | 36 (66.7) |
| Seldom | 8 (22.2) | 10 (23.3) | 16 (34.0) | 17 (31.5) |
| Quite often | 0 (0) | 1 (2.3) | 3 (6.4) | 1 (1.9) |
| Very often | 2 (5.6) | 1 (2.3) | 2 (4.3) | 0 (0) |
| *Missing* | *0* | *0* | *1* | *0* |
| **Avoiding going outside certain hours, n (%)** | | | | |
| No | 8 (22.2) | 21 (50.0) | 24 (51.1) | 49 (90.7) |
| Yes | 28 (77.8) | 18 (42.9) | 23 (48.9) | 5 (9.3) |
| *Missing* | *0* | *1* | *1* | *1* |
| **Affected/restricted everyday life, n (%)** | | | | |
| No, not at all | 25 (71.4) | 28 (68.3) | 36 (76.6) | 49 (94.2) |
| Sometimes | 8 (22.9) | 8 (19.5) | 8 (17.0) | 2 (3.8) |
| Often | 2 (5.7) | 3 (7.3) | 2 (4.3) | 0 (0) |
| Almost always | 0 (0) | 0 (0) | 1 (2.1) | 1 (1.9) |
| *Missing* | *1* | *2* | *2* | *4* |
| **Worried family, n (%)** | | | | |
| No, not at all | 9 (26.5) | 20 (48.8) | 16 (34.0) | 22 (40.7) |
| Seldom | 5 (14.7) | 5 (12.2) | 7 (14.9) | 12 (22.2) |
| Sometimes | 2 (5.9) | 5 (12.2) | 14 (29.8) | 13 (24.1) |
| Almost always | 3 (8.8) | 1 (2.4) | 9 (19.1) | 6 (11.1) |
| *Missing* | *2* | *2* | *3* | *2* |
| Note: Data for answers “don´t know”, or “don’t want to answer” in each question are not presented. Only four participants in the Swedish-born group identified themselves with other gender than female or male and are therefore excluded from the table. | | | | |

**Figure S1** Description of participants worst experience of violence, themes and categories.

**Questionnaire on ViPP**

| **1. Serial number** | | | | | | |
| --- | --- | --- | --- | --- | --- | --- |
| **2. Is it safe for children and young people to move around in the neighbourhood where you live?** | | | | | | |
| Always  Most of the time  Seldom  Never  Don’t want to answer  Don’t know | | | | | | |
| **3. Do you ever go to other areas where children and young people are not safe?** | | | | | | |
| Never  Seldom  Quite often  Very often  Don’t know  Don’t want to answer | | | | | | |
| **4. Do you ever refrain from going out because you are afraid of being attacked, robbed or otherwise victimised? (this includes abuse, harassment or anything else that you consider to be violence)** | | | | | | |
| Never  Seldom  Quite often  Very often  Don’t know  Don’t want to answer | | | | | | |
| **5. Do you avoid going out at certain times of the day for fear of being exposed to violence?** | | | | | | |
| Yes. What time of day is it? | | | | | | |
| No  Don’t know  Don’t want to answer | | | | | | |
|  | | | | | | |
| In the next section, the questions follow a regular pattern about violence you may have experienced or been at risk of experiencing IN SWEDEN. By violence we mean physical, psychological or sexual violence. | | | | | | |
|  | | | | | | |
| **6. To your knowledge, has a friend or acquaintance of yours ever been subjected to physical, psychological or sexual violence in a public place in Sweden? By public environment we mean places outside your or someone else's home, and which are not private. For example, streets, squares, public transport, schools, parks, social media or forums.** | | | | | | |
| Never  Seldom  Quite often  Very often  Don’t know  Don’t want to answer | | | | | | |
| **7. Have you ever experienced physical, psychological or sexual violence in a public place in Sweden? By public places we mean places outside your or someone else's home, and which are not private. For example, streets, squares, public transport, schools, parks, social media or forums.** | | | | | | |
| Never  Seldom  Quite often  Very often  Don’t know  Don’t want to answer | | | | | | |
| If you don't mind, you can tell us what happened: | | | | | | |
|  | | | | | | |
| If YOU have never experienced violence in a public place: skip to question 14. Otherwise, continue with the next question. | | | | | | |
|  | | | | | | |
| **8. If you have experienced violence in a public setting, in which setting was it? Several options can be given.** | | | | | | |
| In a shop, shopping centre, mall or similar  In a café, restaurant, pub or similar  In a park, square, street, car park or similar  On or near public transport  At school, college, university or similar  In the workplace  On or near a leisure activity (including changing rooms)  In the area where you live  On social media or other forums  Don't know  Don't want to answer  Have you experienced violence in other places than those mentioned? Please tell us which ones. | | | | | | |
| **9. If you know or think you know, by whom(s) were you victimised? Several options can be given.** | | | | | | |
| One or more family member  One or more friends  One or more current/former boyfriends or girlfriends, or someone I dated  One or more schoolmates, workmates or similar  One or more school staff, or leaders in extracurricular activities  One or more people unknown to me  By someone else  Don’t know  Don’t want to answer  If you answered by someone else: who was it? | | | | | | |
|  | | | | | | |
| Now there are more detailed descriptions of the types of violence that young people can experience in public spaces. | | | | | | |
|  | | | | | | |
| **10. Have you ever been treated or interacted with in a way that made you feel humiliated in a public setting in Sweden?** | | | | | | |
| Never  Seldom  Quite often  Very often  Don’t know  Don’t want to answer | | | | | | |
| **11. Was the offence or treatment related to any of the following? More options can be given.** | | | | | | |
| Your age  How you dress  The subculture you belong to  Your appearance  Who you socialise with  The area you live in  Your language skills  Your disability  Your gender  Your gender identity and/or expression  Your sexual identity  Your skin colour  Your ethnicity  Your culture/religion  Other  Don’t know  Don’t want to answer  Have you experienced anything else besides the above or is there anything else you would like to share? Please tell us. | | | | | | |
| **12. In a public setting in Sweden, have you ever experienced the following** | | | | | | |
|  | Never | Seldom | Quite often | Very often | Don’t know | Don’t want to answer |
| Physical violence |  |  |  |  |  |  |
| Sexual violence |  |  |  |  |  |  |
| Psychological violence |  |  |  |  |  |  |
| Verbal violence |  |  |  |  |  |  |
| Witnessed violence against someone(s) close to you |  |  |  |  |  |  |
| Witnessed violence against someone(s) not close to you |  |  |  |  |  |  |
| Other violence than above |  |  |  |  |  |  |
| If you answered, ‘other violence’, what kind of violence was it? Please tell us. | | | | | | |
| **13. If you are comfortable doing so, please tell us about the worst experience of violence you have had in a public setting in Sweden.** | | | | | | |
| **14. Is your daily life affected or limited by violence or a risk of violence?** | | | | | | |
| No, not at all  Sometimes  Often  Almost always  Don’t know  Don’t want to answer | | | | | | |
| **15. If you answered ‘yes’ to the previous question, please indicate the ways in which your daily life is affected or limited due to the risk of violence:** | | | | | | |
| What places I go to  What times I go out/do things  Whether I go out/do things alone or with someone  What clothes I wear  What things or activities I do  How I get around  Avoiding certain people/groups of people  Not telling others certain things about myself  Other. For example, it could be that… | | | | | | |
| **16. If you answered that your daily life is affected or limited because of the risk of being subjected to violence. What do you think needs to be done to make your life less affected? You can choose several options.** | | | | | | |
| There should be more adults where children and young people are, so that it is safer  Parents and other adults should learn more about children's and young people's lives  There should be a better image of the area where I live, so that people understand that it is not so dangerous.  We need to reduce violence in the area where I live  We need to teach adults about how the internet works and what children and young people do there, so that they are not so worried  We need to reduce violence on social media and in forums  We need better surveillance by police officers, security guards or cameras  Other. What should be done? | | | | | | |
| **17. Does someone/someone in your family worry that you will suffer violence when you are out/doing things outside the home?** | | | | | | |
| No, not at all  Seldom  Sometimes  Almost always  Don’t know  Don’t want to answer | | | | | | |
| **18. If you answered that your parents/family are worried about you being exposed to violence, how do you see it?** | | | | | | |
| I fully agree. There is reason to be concerned about violence.  I partly agree. Sometimes there is reason to be concerned about violence.  I often think otherwise. There is little cause for concern.  I think completely differently. There is no reason to worry.  Don’t know  Don’t want to answer | | | | | | |
| **19. Whether or not you have experienced violence yourself, would you like to tell us more about what should be done to protect children and young people from being exposed to violence in public places?** | | | | | | |
| **20. Are there other things you think are important for us to know?** | | | | | | |
